# Simulating carbon capture by enhanced weathering with croplands: an overview of key processes highlighting areas of future model development

**DOI:** 10.1098/rsbl.2016.0868

**Published:** 2017-04-05

**Authors:** Lyla L. Taylor, David J. Beerling, Shaun Quegan, Steven A. Banwart

**Affiliations:** 1 Department of Animal and Plant Sciences, University of Sheffield, Sheffield S10 2TN, UK; 2 School of Mathematics and Statistics, University of Sheffield, Sheffield S10 2TN, UK; 3 School of Earth and Environment, University of Leeds, Leeds LS2 9JT, UK

**Keywords:** enhanced weathering, soil acidification, numerical modelling, carbon sequestration, climate change

## Abstract

Enhanced weathering (EW) aims to amplify a natural sink for CO_2_ by incorporating powdered silicate rock with high reactive surface area into agricultural soils. The goal is to achieve rapid dissolution of minerals and release of alkalinity with accompanying dissolution of CO_2_ into soils and drainage waters. EW could counteract phosphorus limitation and greenhouse gas (GHG) emissions in tropical soils, and soil acidification, a common agricultural problem studied with numerical process models over several decades. Here, we review the processes leading to soil acidification in croplands and how the soil weathering CO_2_ sink is represented in models. Mathematical models capturing the dominant processes and human interventions governing cropland soil chemistry and GHG emissions neglect weathering, while most weathering models neglect agricultural processes. We discuss current approaches to modelling EW and highlight several classes of model having the potential to simulate EW in croplands. Finally, we argue for further integration of process knowledge in mathematical models to capture feedbacks affecting both longer-term CO_2_ consumption and crop growth and yields.

## 1. Introduction

Enhanced weathering (EW) is a ‘negative emissions technology’ receiving increasing attention because the Intergovernmental Panel on Climate Change's recently adopted 2°C target may be difficult to attain by reducing fossil fuel emissions alone [1]. Natural chemical weathering is a small sink for atmospheric CO_2_, consuming only approximately 0.25 PgC yr^−1^ [2], with little effect on climate within our lifetimes. This is partly because many soils in warm, humid locations, where weathering should be favoured, are depleted in primary minerals. EW strategies aim to increase CO_2_ consumption by adding silicate rock dust to soils in order to strengthen this sink for atmospheric CO_2_, thus offsetting anthropogenic carbon emissions from fossil fuels.

Mass balance [3,4] and kinetics [5] calculations and a numerical modelling study [6] all suggest that terrestrial EW strategies could contribute to climate change mitigation by consuming significant atmospheric CO_2_. Agricultural land, comprising 15 Tm^2^ globally in 2000 [7], may be particularly suited for EW due to its ease of access and a range of ancillary benefits [8]. Indeed, basalt dust is increasingly suggested as an agricultural amendment on highly weathered nutrient-poor acidic soils in Brazil [9], where the climate is ideal for EW.

The potential of EW to consume CO_2_ or provide co-benefits is difficult to quantify at a range of scales [10]. For example, the reliability of models for long-term forestry planning depends on input weathering rates [11], but weathering rate estimates vary greatly depending on how they are quantified. Several independent weathering rate estimates are therefore recommended when predicting the outcome of EW or assessing its effects [12]. These include empirical estimates based on different types of field data [12], upper CO_2_ consumption limits based on mass balance approaches including assessments of downstream effects such as cation flux in surface water drainage from catchments [3], and rates from process-based numerical models (tables 1 and 2 [6, 13–21, 24]).

Currently, few models provide weathering estimates or predict co-benefits for stakeholders considering deployment of agricultural EW on any scale. However, different classes of models have already been developed to understand plant–soil–biogeochemical interactions, and they perform at least a subset of the calculations needed for EW (tables 1 and 2). With further development, they could provide a diverse set of tools for EW planning and assessment. This is particularly desirable given the increasing focus on multi-model ensembles for estimating uncertainties in model outputs, including water and carbon cycling [25,26] and crop responses to climate change [27–29].

**Table 1.** Overview of models discussed in the text. APSIM, agricultural production systems simulator; GHG, greenhouse gas; ICZ, integrated critical zone; MAGIC, model of acidification of groundwater in catchments; SAFE, soil acidification in forest ecosystems; SOM, soil organic matter.

model	ICZ	Sheffield	WITCH	PROFILE	SAFE	MAGIC	APSIM	DayCent-Chem
dynamic^a^	yes	yes	yes	no	yes	yes	yes	yes
typical scale of usage	site	global	regional or catchment	site or catchment	site or catchment	catchment	site	catchment
driven by	site data	DGVM output	DGVM output	site data	site data	site data	site data	site data
crop growth processes^a^	yes	no	no	no	no	no	yes	yes
GHG production	yes	no	no	no	no	no	N_2_O,CO_2_	N_2_O,NO_x_,CO_2_
soil hydrology^b^	Richard's equation	water balance	input for each layer	input for each layer	input for each layer	input for each layer	bucket or Richard	Richard's equation
SOM dynamics calculated^a^	yes	no	no	no	no	no	yes	yes
soil chemistry calculated^b^	yes	yes	yes	yes	yes	yes	pH	yes
weathering rates^b^	kinetics	kinetics	kinetics	kinetics	kinetics	input	none	input
CO_2_ consumption^b^	yes	yes	yes	no	no	no	no	no
enhanced weathering^b^	planned	yes	no	no	no	no	no	no
references	[13,14]	[6]	[15–17]	[18,19]	[20]	[21]	[22,23]	[24]

^a^Recommended. ^b^Required for cropland EW.

**Table 2.** Additional model details. DON, dissolved organic nitrogen.

model	ICZ	Sheffield	WITCH	PROFILE	SAFE	MAGIC	APSIM	DayCent -Chem
soil texture uniform or by layer	by layer	uniform	by layer	by layer	by layer	by layer	by layer	by layer
soil texture dynamic^a^ or static	dynamic	static	static	static	static	static	static	static (bulk density dynamic)
number of layers	input	10	6 + 1, or 3 (B-WITCH)	input	input	1–2	input	up to 10 + organic + groundwater
release of nutrients from SOM^a^	nutrient and SOM pool-specific	equilibrium	equilibrium	input, and negative uptake of N	input, and negative uptake of N	input	none	rate laws (implicit for Ca, Mg, K)
plant nutrient pools^a^	leaves, wood and roots	implicit	none	implicit	stems, branches, bark, fine roots	whole plant	root, stems, leaves, sucrose, other	shoots, roots
SOM pools^a^	humus, resistant, active	lumped	none	lumped	lumped	one pool	humus, surface residues	active, passive, slow
root depth distribution^a^	uniform	exponential	none	upper layers	input by layer	n.a.	dynamic root growth	input by layer
rhizosphere chemistry^a^	yes	yes	no	no	no	no	no	no
microbes represented^a^	two bacterial guilds	no	no	no	no	no	yes	yes
mycorrhizal fungi^a^	yes	yes	no	no	no	no	no	no
productivity calculated^a^	yes	no	no	no	no	no	yes	yes
nutrient demand based on…^a^	NPP	input NPP	input GPP	input	input	input time series	input ash alkalinity	NPP
nutrients taken up by plants^a^	N, P, K, Ca, Mg	Ca^2+^, Mg^2+^, K^+^, lumped N, 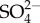 , lumped P	CaMgKSP	N, lumped CaMgK	lumped CaMgK	N	lumped base cations	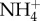 , 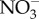 , P, S (CaMgK implicit)
*in situ* mineral surface area per unit land area	empirical function (as PROFILE) of dynamic texture	function of texture and lithology	empirical function of texture (as PROFILE)	empirical function of texture	empirical function of texture (as PROFILE)	implicit	n.a.	none
applied dust mineral surface area per unit land area^b^	(planned) shrinking sphere	shrinking sphere with correction factor	n.a.	n.a.	n.a.	n.a.	n.a.	n.a.
cation exchange capacity^a^	site and layer data	none	fixed for layers 1–4; extrapolated for deeper layers	site and layer data	site and layer data	site data	n.a.	specified or related to an equilibrium phase or kinetic reactant
ion-exchange calculations^a^	Fick diffusion Law (as PROFILE)	none	Fick diffusion Law (as PROFILE)	Fick diffusion Law	Fick diffusion Law (as PROFILE)	Gaines–Thomas equilibria and Langmuir (SO_4_)	n.a.	specified or related to an equilibrium phase or kinetic reactant
exchangeable species^a^	Ca^2+^, Mg^2+^, K^+^, Na^+^, Al^3+^, kinetic adsorption of 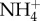	none	Ca^2+^, Mg^2+^, K^+^	base cations, Al^3+^, H^+^	base cations, Al^3+^, H^+^	base cations, Al^3+^, SO_4_^2−^	n.a.	input
mineralogy (*in situ*)	site and layer data	lithological map, uniform	site and layer data	site and layer data	site and layer data	n.a.	n.a.	input
phosphate mineral weathering kinetics^a^	yes	no	yes	yes	yes	n.a.	n.a.	n.a.
carbonate/sulfate mineral weathering/chemistry^a^	equilibria	equilibria	kinetics	none	none	n.a.	n.a.	input annual flux
fertilizer^a^	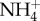 fertilizer	no	no	with deposition	with deposition	with deposition	N, P from manure or fertilizer, residues	N, P, S organic or inorganic fertilizer
deposition^a^	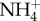 , 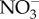 , K^+^, low molecular weight N, P	none	none	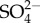 , NO_x_, 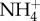 , 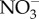 , NH_3_, base cations	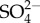 , 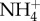 , 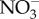 , lumped CaMgK	Ca^2+^, Mg^2+^, K^+^, Na^+^, 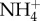 , 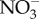 , 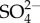 , Cl^−^, F^−^	none	wet and dry H^+^, Ca^2+^, Mg^2+^, K^+^, Na^+^, Cl^−^, 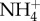 , 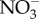 , 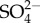
pyrite dissolution	none	specified based on lithology	input	none	none	input	none	input annual flux
secondary minerals^a^	gibbsite equilibria	equilibria only	kinetics	gibbsite equilibria	gibbsite equilibria	aggregated Al(OH)_3_ equilibria	none	input annual flux
weathering agents^b^	H^+^, CO_2_, H_2_O + organic ligands	oxalate, H^+^, H_2_O, OH^−^	H^+^, H_2_O, OH^−^, ligands	H^+^, H_2_O + organic ligands, CO_2_	H^+^, H_2_O + organic ligands, CO_2_	n.a.	none	n.a.
silicate weathering^b^	kinetics	kinetics	kinetics	kinetics	kinetics	Bayesian model with input fluxes per unit land area	no	input annual flux
N species^b^	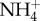 , 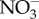	implicit	implicit	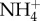 , 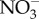	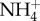 , 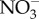	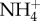 , 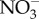	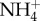 , 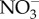	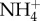 , 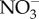 , N_2_O, NO_x_, N_2_, DON
N immobilized by microbes^a^	yes	no	no	net uptake	yes	yes	no	yes
nitrification^a^	kinetic	no	no	kinetic	kinetic	input	yes	yes
denitrification	yes	no	no	no	no	input	yes	yes
volatile NH_3_	yes	no	no	no	no	no	no	yes
N leaching^a^	NO_3_^−^	no	no	no	no	no	NO_3_^−^	yes
Al species	13 species	lumped	Al^3+^, AlOH^2+^, Al(OH)_2_^+^	Al^3+^, AlOH^2+^, Al(OH)_2_^+^	Al^3+^, AlOH^2+^, Al(OH)_2_^+^	13 species	none	input
P species	PO_4_^3−^	lumped	H_3_PO_4,_ 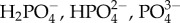	none	none	none	lumped	input
organic acids	lumped trivalent	lumped oxalate	lumped ligands	lumped monovalent	lumped monovalent	lumped triprotic	none	lumped triprotic

^a^Recommended.

^b^Required for cropland EW.

## 2. Calculating the weathering sink for atmospheric CO_2_

When atmospheric CO_2_ dissolves in water at pH values above the acid endpoint for H_2_O–CO_2_ proton balance, it forms carbonic acid that dissociates and lowers the pH of water, favouring mineral dissolution (weathering). As weathering of oxide minerals such as cation-bearing silicates progresses, the oxide ion acts as a strong base and consumes protons. The reaction releases a molar charge equivalent of base cations to solution and this increase in alkalinity raises pH; more CO_2_ dissolves (is *consumed*) due to the increased solubility of inorganic carbon ions 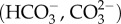 at elevated pH in equilibrium with soil *p*CO_2_ (gas).

Absolute weathering rates are calculated with mineral-specific rate laws capturing their dependence on reactive dissolved species such as H^+^, and may vary over several orders of magnitude depending on mineralogy, temperature, pH and solute concentrations. Both protons and hydroxide ions are potent weathering agents, and rates are generally lowest at circumneutral pH where both ions are at relatively low molar activity. Rates increase at lower and higher pH as H^+^ or OH^−^ activity increases. Weathering mechanisms dominating at near-basic and higher pH can be important in arid to semi-arid climates, but have negligible effect in humid climates where pH is normally much lower. Solute concentrations and pH depend on hydrology and flushing rates along with weathering rates and nutrient cation and anion cycling. Existing numerical weathering models [6,15,20] calculate weathering and flushing rates and soil water chemistry, but require hydrological and soil texture parameters along with mineralogy and temperature.

Acidity, alkalinity and pH are strongly influenced by base cations and their removal rate by hydrological flushing. The source strength for base cations (mol ha^−1^ y^−1^) arises by multiplying mineral-specific weathering rates (mol m^−2^ mineral s^−1^) by reactive mineral surface area (m^2^ ha^−1^ or m^2^ m^−3^ soil). Reactive surface area is, therefore, a critical parameter in determining weathering rates and pH, usually estimated with empirical functions based on soil texture and mineralogy [6,20]. EW models must account for changing surface area as the applied particles dissolve; one method is to assume these particles are shrinking spheres [6,30].

Soil chemistry also depends on the *in situ p*CO_2_ that suppresses pH and arises from microbial mineralisation of soil organic matter (SOM). SOM, largely derived from decaying vegetation [31], is a carbon and energy source supporting communities of respiring organisms that also take up nutrient ions. SOM and microbial activity also affect soil structure and the related moisture retention and water permeability. Because soil pore space architecture and water retention slow upward diffusion of respired CO_2_ in soils, respiration causes order-of-magnitude increases in soil CO_2_ levels beyond those of the atmosphere. Although degassing will occur when CO_2_-charged soil drainage water reaches rivers, within the soil profile elevated CO_2_ accelerates weathering through increased H^+^-activity.

## 3. Agricultural soil chemistry depends on nitrogen cycling

Agricultural practices have a profound impact on the water, carbon and nitrogen [32] cycles, and by altering soil chemistry can create acidic, nutrient-depleted conditions affecting weathering regimes and crop growth. Nutrients in harvested plant material cannot enter SOM pools or be mineralised to release alkalinity. Soil acidification leads to Al and Mn toxicity [33], limiting nutrient uptake, root growth and crop yields, so alleviation of this global agricultural problem is, therefore, a potential EW co-benefit.

Plants acidify soil by several mechanisms. For example, roots exude low-molecular-weight organic acids providing protons at soil pH values high enough for the acids to be dissociated, and the anions (oxalate/citrate/malate) are chelating ligands accelerating weathering [34]. However, nutrient demand is the main source of acidification from living plants, because protons are exchanged for nutrient cations in the ‘rhizosphere’ soil pore waters surrounding roots and mycorrhizal hyphae [35].

Rhizosphere acidification occurs due to cation uptake in excess of anion uptake, which depends on availability of nitrate (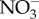) and ammonium (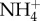). Decomposition of organic N in SOM produces ammonium [35], which is converted to nitrate at a rate depending on soil pH, temperature, moisture and the existing nitrate and ammonium concentrations [36]. Oxidation of organic N to nitrate is accompanied by proton release and acidification of soil. Ammonium may be retained on cation exchange surfaces and on cell walls in the apoplast [37], but nitrate is highly mobile and prone to transport from the soil profile. Because of these transformations, application of ammonium or organic fertilizers also contributes to nitrification and generation of protons remaining in the soil following nitrate leaching [35]. These protons reduce the solubility of inorganic carbon ions, leading to undersaturated pore waters with respect to dolomite (CaMg(CO_3_)_2_) and calcite (CaCO_3_). Dissolution of these ‘liming’ minerals can then release CO_2_ to the atmosphere [38].

Legumes with symbiotic nitrogen-fixing bacteria in root nodules are less likely to take up nitrate. They generate protons and exchange these for base cations from the soil solution, but the extent of rhizosphere acidification depends in part on the forms of the organic acids produced during carbon assimilation and is therefore species-specific [35].

Because nutrient demand, fertiliser usage, root distributions and rhizosphere processes are crop-specific, proper representation of crop rotations will be critical for capturing EW effects. Teixeira *et al.* [39] showed that modelled soil nitrogen differed substantially between four simulation methods for wheat, maize and kale rotations.

Models simulating soil chemistry and EW in croplands need to capture the most important processes of the nitrogen cycle, including mineralisation, nitrification and nutrient uptake by plants and microbes. These comprise the four major nitrogen transformations in agricultural ecosystems [32]. Key loss pathways are nitrate transport in drainage waters, harvests and NH_3_ volatilization, while denitrification, burning, erosion and trace gas (N_2_O, NO*_x_*) fluxes are often smaller [32].

## 4. Adapting existing models to simulate enhanced weathering in croplands

The need to assess potential management strategies for remediating soil acidification has driven the development of nutrient cycling modules within crop models. A well-established example, APSIM [40], simulates soil pH based on proton budgets, calculating ‘excess’ cation uptake along with carbon and nitrogen cycle imbalances due to removal of biomass, organic matter accumulation and leaching of organic anions. APSIM also calculates crop growth and soil water cycling. This model ignores both cation exchange and weathering [41], but if these were incorporated it could provide detailed predictions of CO_2_ consumption during crop growth because it considers changes in multiple plant alkalinity pools with time. APSIM can simulate management practices including crop rotations [39], most of the major nitrogen-cycling processes [22] and greenhouse gases (GHGs) including N_2_O [42]. Crop models such as APSIM are, therefore, already well placed to inform emissions policy [43].

Another example, DayCent [44], simulates a similar range of processes [43] and has been combined with the aqueous geochemistry model pH redox equilibrium C (PHREEQC) [45] to simulate GHG fluxes and stream water chemistry in forested and alpine catchments [46]. Although computationally intensive [24], DayCent-Chem requires input weathering fluxes and ignores base cation cycling, assuming uptake is balanced by release from SOM. Nevertheless, with further development this integrated model could be a powerful predictive tool for EW, even capturing the fate of heavy metals that might be released from the applied dust [47].

Acid rain and resulting S and N deposition drove the development of policy-relevant [48] models to estimate the ‘critical loads’ of acid that ecosystems can tolerate without damage; linkages to plant or biodiversity models have allowed calculation of soil chemistry thresholds for different vegetation types [49]. These models typically include equilibria with solid phases, but often require initial weathering rate and nutrient uptake data as inputs. A widely used dynamic example is MAGIC [50], which calculates changes in soil chemistry with time at catchment scale. MAGIC includes carbonate equilibria, carbon and nitrogen cycling, and adjusts the input exchange capacities and weathering rates to match observed calibration data.

PROFILE is a steady-state soil chemistry model [18,19] with a long history of use in forestry and agriculture, and its dynamic version, SAFE [20], calculates changes in acid–base balance over time. These models have been employed at field and catchment scale and could easily calculate CO_2_ consumption due to EW by incorporating appropriate rate and surface area expressions for the applied minerals.

Several process-based weathering models calculate CO_2_ consumption and weathering at regional or global scale given climate, soil water flow and respiration, lithology and a suite of weathering rate laws. One such model, WITCH [15], has been applied to large watersheds such as the Mackenzie River basin [16]. Another, the less computationally complex Sheffield weathering model [6], has previously been employed globally in a non-agricultural EW context. These models rely on the output of dynamic global vegetation models (DGVMs) simulating plant productivity, soil respiration and hydrological flows and could be adapted to assess EW in agricultural practices at farm scale.

A recently developed code to assess the interaction of soil structure with vegetation and mineral weathering is the Integrated Critical Zone (ICZ) model incorporated into the Soil and Water Assessment Tool software package [51]. The ICZ model simulates the plant–soil–water system with one-dimensional water flow and reactive transport in the soil profile. It incorporates a vegetation model, SOM dynamics, nutrient transformations of N and P, mineral weathering kinetics based on the SAFE code noted above, and geochemical speciation equilibria between solutes, mineral and gas phases and ion-exchange surfaces [13,14]. The ICZ includes a sub-model for changes in soil structure with changing biological activity, organic carbon inputs and mineralization, and the resulting changes in bulk soil hydraulic and transport parameters feedback to the equations describing hydrological flow and transport processes. Simulating EW with the code still requires process descriptions accounting for changing reactive surface area as rock powder dissolves; e.g. shrinking core equations.

## 5. Capturing dynamic plant–soil-enhanced weathering interactions

Weathering depends on productivity, hydrology and nutrient cycling, but EW-type treatments have been shown to affect plant growth [52] and water cycling [53] over several years, and phosphorus availability within several months [54]. These feedbacks indicate that EW should be coupled to modules calculating crop growth, nutrient cycling and soil hydrology.

Models integrating EW with crop productivity and nutrient limitation [55] are especially desirable. Although some DGVMs have been adapted for food and biofuel crops [22], they represent nutrient cycling to a varying degree [56], limiting their ability to predict productivity under climate change [55]. Likewise, crop models explicitly representing nitrogen stress [57] suggest it will be significant for food crops as climate changes. Phosphorus limitation, a common problem in highly weathered tropical soils, is difficult to quantify but is being incorporated into DGVMs [58], albeit without weathering kinetics. However, P is derived from weathering, and P cycling deserves priority because basalt soil amendments increase available soil P with a likely feedback on crop productivity [54].

Separate plant and SOM pools for each nutrient along with root and nutrient uptake dynamics [59] would allow calculation of changing chemistry as a crop matures. Non-structural nutrients such as potassium may be released from the fastest-decomposing litter pools, while nitrogen may be retained in more recalcitrant SOM pools.

EW might also change the soil properties governing hydrology within DGVMs. Overall mineral composition and particle size distribution of the applied material change with time because of the different weathering rates of the constituent minerals. Soil porosity may change following application if individual or aggregated [60] particles change micropore characteristics; particles may be flushed to deeper layers or lost in run-off when they become smaller than micropores. Particle transport is, therefore, a priority for development and inclusion in EW models with associated hydrologic feedbacks on crop production.

## 6. Conclusion

EW models adopted for croplands need to capture the major water, nutrient and GHG fluxes to realistically simulate CO_2_ capture by weathering. These fluxes do not occur in isolation; they interact and lead to feedbacks across a range of timescales. Critically, water flow and the concentration of weathering agents such as H^+^ in the soil solution are strongly controlled by crops. When combined with agricultural practices, these processes can lead to persistent soil acidity, suggesting a co-benefit for farmers because the base cations released by EW increase the alkalinity of the soil.

Existing crop models typically ignore weathering, but may simulate nitrogen cycling, proton balance and GHGs. On the other hand, weathering models driven by coupling with DGVMs may not capture enough cropland processes. The ICZ model couples a vegetation model with mineral solubility for carbonate phases and silicate mineral weathering kinetics. Nevertheless, a diverse set of modelling tools of varying complexity and taking a variety of approaches would likely be more useful for assessing the uncertainties and relative importance of EW parameters and processes than any one model.

One of the most important criteria for quantifying EW dynamics that emerges from this review of modelling approaches is representing the feedbacks between crop growth and mineral weathering kinetics. Understanding the mechanistic process linkages between EW and climate mitigation requires coupled calculations, on intra-annual time scales of relevance for agricultural practices and on inter-annual time scales for climate change mitigation. A priority for the development and assessment of EW across all scales as a possible climate mitigation option is to develop this coupled modelling capability and validate it with appropriate field and laboratory experiments.
